# Advances in Morphology Control of Through-Glass via Wet Etching for High-Performance Glass-Based Packaging

**DOI:** 10.3390/mi17091100

**Published:** 2026-09-20

**Authors:** Qi Zhang, Houtong Liu

**Affiliations:** 1 School of Microelectronics & Data Science, Anhui University of Technology, Ma’anshan 243002, China; qizhang@ahut.edu.cn; 2 Anhui Huachuang Hongduo Optoelectronic Technology Co., Ltd., Hefei 230000, China

**Keywords:** through-glass via (TGV), wet etching, morphology control, morphology evolution, sidewall roughness, laser-induced selective etching, TGV fabrication

## Abstract

Through-glass vias (TGVs) are important high-aspect-ratio structures for glass-based advanced integration, and their wet-etching morphology significantly influences metallization quality, electrical performance, and reliability. This review establishes a process–morphology–performance framework to analyze morphology control strategies in TGV wet etching. Existing approaches are classified according to their morphology regulation mechanisms, including conventional hydrofluoric acid/buffered hydrofluoric acid etching, material modification, assisted etching, laser-induced selective etching, and hybrid strategies. The effects of these approaches on aspect ratio, sidewall roughness, sidewall verticality, dimensional uniformity, and defect formation are systematically discussed. Conventional HF/BHF etching exhibits high process maturity and low cost but is limited by isotropic dissolution and mass transport effects. Material modification and assisted etching improve local etching selectivity but remain dependent on glass composition and processing conditions. Laser-induced selective etching provides enhanced morphology control through localized structural modification. Future TGV fabrication requires integrated regulation of material modification, mass transport, and process monitoring to achieve precise via morphology optimization. This review provides guidance for selecting and optimizing wet-etching strategies for high-quality glass microstructure fabrication.

## 1. Introduction

Driven by the advancement of high-performance computing, artificial intelligence, millimeter-wave communications, and heterogeneous integration systems, advanced packaging is evolving from traditional single-chip architectures toward 2.5-D/3-D integration, chiplet integration, and system-in-package solutions. Three-dimensional integration enhances integration density via vertical interconnections while offering novel system architecture options for heterogeneous integration across disparate device technologies [1]. Furthermore, 2.5-D and 3-D heterogeneous integration platforms utilize interposers, embedded bridges, and high-density die-to-die interconnections to shorten interconnect length, boost bandwidth density, and reduce power consumption [2,3]. However, the performance of TGV-based interconnects depends not only on the successful formation of through-holes but also on precise control of via morphology, including aspect ratio, sidewall roughness, sidewall verticality, and dimensional uniformity.

Against the backdrop of continued miniaturization and increasing integration density in microelectronic systems, growing attention has been paid to the reliability of metallic interconnects and the early detection of packaging defects. Studies on electromigration, wire-bonding technologies, and electrical-parameter-based damage detection have shown that interconnect materials, interface conditions, and internal structural damage can significantly affect the long-term stability of packaged devices [4,5,6].

In this context, glass interposers and glass-core substrates are considered compelling alternatives to silicon interposers and organic substrates due to their low dielectric loss, superior dimensional stability, tunable coefficient of thermal expansion (CTE), and potential for panel-level large-area processing [7,8,9,10,11]. Through-glass vias (TGVs) serve as the core structural element for achieving vertical interconnections in glass-based packaging, performing a role analogous to through-silicon vias (TSVs) in silicon-based 3-D integration. Compared to silicon substrates, glass substrates exhibit excellent electrical insulation, low dielectric loss, and tailorable CTE, making them particularly well-suited for high-frequency, low-loss, and large-area packaging applications. Delmdahl and Paetzel early discussed high-density TGV laser drilling from a 2.5-D/3-D packaging perspective, highlighting that high-density via fabrication requires simultaneous consideration of via diameter, edge damage, processing throughput, and subsequent metallization compatibility [12]. Galler et al. demonstrated transitions from monolithic microwave integrated circuits (MMICs) to dielectric waveguides using TGVs and glass structures in glass-based packaging above 150 GHz, further illustrating that the geometric precision, interconnect integrity, and dielectric loss of TGVs directly dictate high-frequency packaging performance [13]. Consequently, TGV fabrication should not be viewed merely as “drilling holes in glass,” but rather as a critical manufacturing link bridging glass materials, micromachining processes, metallization filling, and ultimate device performance.

From the perspective of via-formation methodologies, conventional wet-etching techniques using HF or buffered hydrofluoric acid (BHF) are mature and cost-effective, making them suitable for low-aspect-ratio micro-grooves, micro-cavities, and preliminary via processing. However, due to the amorphous nature of glass, its wet-etching behavior is typically isotropic, which readily induces undercutting, via enlargement, sidewall tapering, and inadequate profile verticality [14,15]. Williams and Muller systematically reviewed the etch rates of common micromachining materials, demonstrating that etch rate, material selectivity, and mask compatibility constitute the foundational parameters for wet-process design [16]. Meanwhile, research by Konstantinova et al. on deep wet etching of fused silica in buffered oxide etchant (BOE) solutions further revealed that even with buffered fluoride systems, mask durability, etch rate, and profile isotropy remain primary bottlenecks in deep-structure microfabrication [17]. Therefore, relying solely on conventional wet etching fails to fulfill the comprehensive technical demands of high-performance TGVs, which require a high aspect ratio, vertical sidewalls, and low sidewall roughness.

To overcome the limitations of isotropic etching, composite processes, such as laser-assisted wet etching, selective laser etching, and laser-induced deep etching, have emerged as crucial development directions in glass micromachining. The underlying concept is to introduce localized modified regions, nanodefects, or stress-damaged zones on the surface or within the bulk of glass, thereby driving the dissolution rate of the modified regions in subsequent HF, KOH, or other etching solutions significantly higher than that of the unmodified matrix to achieve directional selectivity. Previous studies have utilized methods such as laser-induced backside wet etching (LIBWE), laser-induced wet etching (LIWE), and selective laser etching (SLE) to achieve high-aspect-ratio structures with low sidewall roughness [18,19,20]. Meanwhile, laser-induced deep etching (LIDE), functioning as a two-step process of “laser modification followed by wet etching,” has been applied to high-precision TGV, glass micro-structure, and high-frequency glass device fabrication; relevant investigations demonstrate that this technique can form high-aspect-ratio microstructures in thin glass while suppressing defects such as microcracks, chipping, and thermal damage [21,22].

In recent years, LIDE, SLE, and femtosecond laser-assisted wet etching have further expanded from TGV via formation to the fabrication of optical waveguides, display glass, micro-optical elements, and functionalized glass surfaces, highlighting the strong scalability of such hybrid processes in processing complex glass microstructures [22,23,24,25].

Although numerous studies have investigated individual TGV fabrication approaches, including wet etching mechanisms, laser-assisted processing, and process optimization, existing reviews mainly focus on specific fabrication routes or etching systems. The relationship between etching strategies, morphology evolution, and subsequent packaging performance has not been systematically established.

However, several challenges remain for high-performance TGV manufacturing. On one hand, high-aspect-ratio TGVs typically demand prolonged etching duration, which intensifies problems such as internal etchant concentration gradients, localized etchant depletion, and reaction byproduct removal; conversely, high-throughput manufacturing dictates shortened processing cycles and enhanced array processing efficiency, creating a fundamental trade-off that makes optimizing the process window difficult. On the other hand, morphological features, including TGV sidewall roughness, sidewall verticality, and internal residues, exert a profound influence on seed-layer coverage, copper filling completeness, high-frequency signal loss, and thermo-mechanical reliability [19,26,27]. Therefore, a comprehensive framework linking etching processes, morphology evolution, and final performance is required for understanding and optimizing TGV wet etching.

To ensure the comprehensiveness and objectivity of this review, a literature search was conducted using the Web of Science Core Collection, Scopus, IEEE Xplore, ScienceDirect, and Google Scholar databases. The literature search was initiated in January 2026, with the search strategy based on combinations of keywords including “through-glass via”, “TGV”, “wet etching”, “glass etching”, “morphology control”, “sidewall roughness”, and “verticality”. Relevant studies were selected based on their relevance to TGV fabrication, etching mechanisms, morphology evolution, and process optimization. Studies mainly focusing on silicon TSVs or unrelated glass micromachining processes were not considered.

In contrast to previous reviews focusing mainly on individual fabrication routes or etching systems, this review emphasizes morphology control during TGV wet etching.

Against this backdrop, this review focuses on morphology control during TGV wet etching and establishes a process–morphology–performance framework to correlate etching strategies, morphology evolution, and downstream packaging performance. The fundamental mechanisms of glass wet etching are discussed from the perspectives of chemical reactions, etchant transport, and morphology evolution. Different technological routes, including conventional wet etching, material modification, auxiliary-field-assisted etching, laser-induced selective etching, and hybrid strategies, are analyzed and compared based on their morphology regulation capabilities, advantages, and limitations. Furthermore, the relationships between via morphology, metallization quality, electrical characteristics, and reliability are summarized to clarify the practical significance of morphology control.

## 2. Through-Glass via (TGV) Wet-Etching Mechanisms and Evaluation Metrics

### 2.1. Research Background and Problem Orientation of TGV Wet Etching

TGV fabrication typically encompasses via formation, sidewall quality control, seed-layer deposition, metal filling, and reliability evaluation. Within this workflow, wet etching functions not only as an independent via-formation method, but also as a critical component coupled with laser modification, photosensitive glass exposure, and ultrasonic assistance to remove modified regions, attenuate damage layers, and refine sidewall morphology. Consequently, this paper first discusses why achieving high-aspect-ratio TGVs with vertical sidewalls remains challenging in wet etching processes. The discussion focuses on the coupling effects of etching chemistry, mass transport, and morphology evolution, providing a mechanistic foundation for understanding subsequent technological strategies.

### 2.2. Fundamental Mechanisms of Glass Wet Etching

Glass fundamentally incorporates $SiO_{2}$ as its main network former, alongside network modifiers such as $Na_{2}O$ and CaO, as well as components like $B_{2}O_{3}$ and $Al_{2}O_{3}$ that alter the degree of glass network connectivity. The essence of wet etching lies in chemical reactions between the etchant and the glass network, which cleave Si-O-Si bonds and convert them into soluble products. Depending on the etchant system, TGV-related wet etching is primarily categorized into HF-based etching (acidic) and $OH^{-}$-based etching (alkaline). These two systems differ significantly in reactive species, etch rates, mask compatibility, and final profile morphology.

#### 2.2.1. Acidic Etching: Reaction Pathways and Interfacial Kinetics

HF-based etching is one of the most widely investigated acidic etching approaches for glass micromachining and TGV fabrication. The dissolution behavior of silicon-based glass networks is governed by the interaction between fluoride species and the Si–O–Si structure, where HF and HF_2_^−^ participate in bond cleavage and the formation of soluble fluorosilicate products such as SiF_6_^2−^ [28,29,30,31]. However, the resulting TGV morphology is not determined solely by chemical dissolution. Instead, etching profiles are strongly influenced by the coupled effects of interfacial reaction kinetics, local reactant concentration, by-product transport, and glass network composition. Previous studies have demonstrated that HF and HF_2_^−^ are the primary reactive species involved in silicate glass dissolution, while H^+^ promotes the cleavage of silicon–oxygen bonds and accelerates glass network depolymerization [28]. These factors collectively affect etching anisotropy, sidewall taper, and profile uniformity in high-aspect-ratio glass structures. For glass systems predominantly composed of SiO_2_, the acidic etching reactions can be described by the following stepwise and overall chemical equations:

(1) $${\mathrm{SiO}}_{2}+4\mathrm{HF}\to {\mathrm{SiF}}_{4}(g)+2H_{2}O$$

(2) $${\mathrm{SiF}}_{4}+2\mathrm{HF}\to H_{2}{\mathrm{SiF}}_{6}(aq)$$

The overall reaction is represented as:

(3) $${\mathrm{SiO}}_{2}+6\mathrm{HF}\to H_{2}{\mathrm{SiF}}_{6}+2H_{2}O$$

Equations (1) and (2) describe the intermediate reactions during SiO_2_ dissolution, while Equation (3) represents the overall stoichiometric transformation. In practical TGV etching processes, the final morphology is additionally affected by reactive species concentrations (HF, HF_2_^−^, H^+^), glass composition, surface reaction kinetics, and by-product transport. Therefore, these equations mainly provide the chemical basis for understanding material dissolution rather than directly predicting the final etched profile.

In multicomponent glasses, network modifiers such as $Na_{2}O$, CaO, and MgO introduce non-bridging oxygen structures into the glass network, reducing silicate network connectivity and locally elevating dissolution rates [32]. Concurrently, certain metal ions react with $F^{-}$ to form low-solubility fluorides such as $CaF_{2}$, $MgF_{2}$, and $AlF_{3}$, which can induce localized passivation and alter the resulting etching morphology. This indicates that glass composition determines not only the etch rate, but also the isotropic or quasi-isotropic dissolution behavior that ultimately governs TGV taper, sidewall quality, and surface morphology.

From a kinetic perspective, the HF etch rate is typically dependent on HF concentration and temperature, which can be described by an empirical rate expression:

(4) $$v=k\cdot C_{\mathrm{HF}}^{n}\cdot \exp\left(-\frac{E_{a}}{RT}\right)$$

where *v* is the etch rate, *k* is the rate constant, $C_{HF}$ is the HF concentration, n is the reaction order, $E_{a}$ is the apparent activation energy. The reaction order n reported in literature generally ranges from 1.2 to 1.8, and the apparent activation energy for the amorphous $SiO_{2}$ phase is approximately 30–40 kJ/mol [28]. It should be clarified that this equation serves as an empirical formulation primarily utilized to describe the effects of HF concentration and temperature on the etch rate under specific conditions; for BOE/BHF or multicomponent glass systems, the actual etch rate is concurrently governed by $HF_{2}^{-}$, $H^{+}$, reaction product deposition, and glass formulation.

#### 2.2.2. Diffusion Limitations and Deep-Via Morphology Evolution in Vias

In addition to the surface chemical reactions described above, deep TGV etching is also affected by etchant diffusion within the via, local concentration variations, and the removal of reaction products. In laser-modification-assisted wet etching, the via structure typically evolves from a non-through laser-modified channel into a through TGV. Before breakthrough, etching non-uniformity is mainly reflected in the difference between the via opening and the via bottom. After breakthrough, the etchant can enter from both sides, and morphological variations are more commonly observed between the surface opening regions and the middle or waist region inside the via. Consequently, high-aspect-ratio TGVs often exhibit cross-sectional profiles in which the surface opening diameter is larger than the via waist diameter, together with tapered sidewalls or non-uniform diameters along the substrate thickness. To evaluate how the competition between surface reaction kinetics and internal mass transport influences TGV profile evolution, the Damköhler number (Da) is introduced as a qualitative descriptor.

(5) $$\mathrm{Da}=\frac{\mathrm{reaction}\;\mathrm{rate}}{\mathrm{diffusion}\;\mathrm{rate}}=\frac{k_{s}L}{D_{\mathrm{eff}}}$$

where $k_{s}$ is the surface reaction rate constant, *L* is the characteristic diffusion length or via depth, and $D_{eff}$ is the effective diffusion coefficient. A low Da value indicates that the etching process is mainly reaction-controlled, whereas a high Da value suggests that mass transport limitations become significant. In deep TGV structures, insufficient etchant replenishment and slow by-product removal may generate concentration gradients between the via opening and central region, resulting in tapered profiles and non-uniform sidewall morphology. Therefore, simply increasing HF concentration cannot indefinitely improve TGV quality, because accelerated surface reactions may coexist with diffusion-induced etching non-uniformity.

In this review, the Damköhler number is therefore used to distinguish reaction-controlled and mass-transport-controlled regimes during TGV etching. Although it does not explicitly account for evolving via geometry, local concentration gradients, or flow conditions, it provides a qualitative understanding of morphology evolution in high-aspect-ratio structures.

To address the limitations of internal etchant diffusion and byproduct removal, existing studies frequently employ forced convection, reciprocal motion of substrates, ultrasonic assistance, pulsed solution supply, and thermal control to promote internal etchant replenishment and accelerate the expulsion of gas bubbles and reaction byproducts. These methods do not inherently alter the amorphous structure of glass; rather, they minimize etch-rate disparities by improving localized etchant exchange and byproduct removal.

To further describe morphology evolution during TGV etching, numerical approaches such as the level set method have been employed to represent the movement of the etching interface [33]. For an isotropic etching front, the conventional level set equation can be expressed as:

(6) $$\frac{\partial \varphi }{\partial t}+V_{n}\left|\nabla \varphi \right|=0$$

where φ represents the level set function and *V_n_* denotes the normal velocity of the etching front. This equation provides a general description of interface evolution driven by local etching rates. Therefore, this equation is used here to illustrate the general mechanism of morphology evolution rather than to quantitatively predict the profile of a specific TGV structure.

#### 2.2.3. Mechanisms and Limitations of Alkaline Etching

Alkaline etching provides an alternative dissolution route for glass micromachining, where the interaction between hydroxide species and the glass network determines the resulting etching behavior. Alkaline etchants such as KOH, NaOH, and tetramethylammonium hydroxide (TMAH) promote glass dissolution through hydroxide-assisted cleavage of Si–O–Si bonds, leading to soluble silicate species. The corresponding network dissolution reactions can be simplified as:

(7) $$\equiv \mathrm{Si}–O–\mathrm{Si}\equiv +{\mathrm{OH}}^{-}\to \equiv \mathrm{Si}–\mathrm{OH}+\equiv {\mathrm{SiO}}^{-}$$

For network structures predominantly composed of $SiO_{2}$, the net reaction can be simplified as:

(8) $${\mathrm{SiO}}_{2}+2{\mathrm{OH}}^{-}\to {\mathrm{SiO}}_{3}^{2-}+H_{2}O$$

Compared with HF-based etching, alkaline dissolution behavior exhibits a stronger dependence on glass composition, solution pH, and processing duration. Wang et al. reported that the dissolution behavior of borosilicate glass in TMAH was strongly influenced by compositional components such as Ca, Zr, Al, and B, which affected the transition between dissolution and precipitation-controlled regimes [34]. Consequently, while alkaline systems can be applied to surface treatment or corrosion behavior studies for specific glass materials, their etch rate, morphology control capabilities, and material applicability remain significantly constrained by glass composition. On the other hand, glass wet etching is typically dominated by acidic systems such as HF or HF/HCl, and amorphous glass generally exhibits isotropic characteristics during wet etching, making it difficult to satisfy the fabrication requirements of high-aspect-ratio TGVs on its own [35]. Therefore, alkaline etching is more suitable as an auxiliary step in specific surface treatments, low-damage processing, or hybrid processes, rather than serving as the mainstream via-formation route for high-aspect-ratio TGV manufacturing.

#### 2.2.4. Physical Essence of Isotropy and Strategies for Breakthroughs

Glass is an amorphous material lacking statistically preferred crystallographic orientations analogous to single-crystal silicon; hence, its wet etching typically exhibits pronounced isotropic characteristics. For glass networks dominated by $SiO_{2}$, HF-based etching primarily involves the cleavage of Si-O-Si networks and the generation of soluble fluorosilicate products such as $SiF_{6}^{2-}$ and $H_{2}SiF_{6}$. Concurrently, etchant diffusion, localized concentration variations, and the evacuation of reaction byproducts jointly dictate the internal via-etch rate. Therefore, isotropy in TGV wet etching is not merely a consequence of the material structure itself, but is also intimately tied to surface reaction kinetics, etchant diffusion, localized concentration gradients, and byproduct removal, which collectively determine the achievable sidewall profile and aspect ratio of TGV structures.

**Figure 1:**
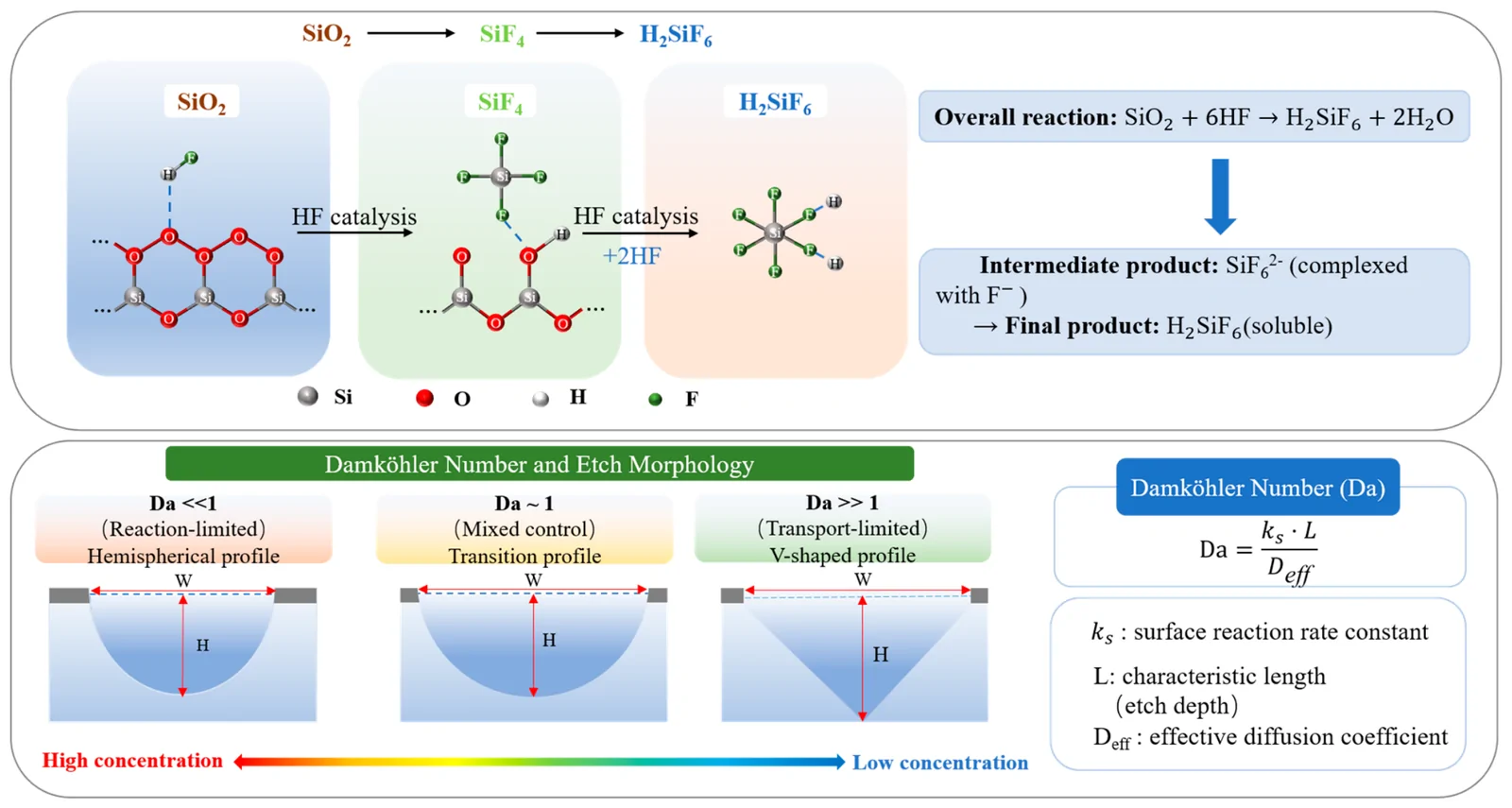
Schematic illustration of glass wet etching mechanism, mass transport effects, and TGV morphology evolution.

Figure 1 schematically illustrates the coupling between chemical reactions, etchant diffusion, and morphology evolution during glass wet etching. The etched profile is determined by the combined effects of HF-induced dissolution reactions and internal etchant transport within the via. At the same time, the etched profile is also affected by the relative rates of surface reaction kinetics and etchant diffusion within the via. The competition between these two factors determines the local dissolution rate distribution along the via depth and consequently influences the final TGV morphology. When the etching process is mainly controlled by surface reaction kinetics, the concentration gradient inside the via remains relatively small, resulting in a relatively uniform dissolution profile. As a result, the profile becomes more susceptible to lateral expansion, insufficient etching in the via waist region, and deviation from an ideal vertical structure.

This mechanism indicates that, in conventional HF/BHF-based wet etching, isotropic etching caused by the amorphous structure of glass and diffusion limitations inside the via jointly affect the etched profile, making it difficult to separate vertical etching from lateral etching. In typical isotropic etching, lateral and vertical material removal increase at nearly the same rate. Therefore, through-via profiles are prone to undercutting, rounding, or tapered shapes, which limit both the aspect ratio and via verticality. This shows that the key to morphology control in TGV wet etching is not simply to increase the etch rate, but to achieve a balance among etching efficiency, etchant renewal inside the via, suppression of lateral etching, and profile verticality.

Therefore, the performance of TGV wet etching should not be evaluated solely on the basis of etch rate or etch depth. A comprehensive assessment should also consider parameters such as aspect ratio, sidewall roughness, via verticality, via diameter uniformity, and defect level. Based on the mechanism discussed above, the following section further summarizes the key parameters commonly used to evaluate TGV wet-etching morphology and process performance.

### 2.3. Key Evaluation Metrics for Etching Morphology and Effectiveness

The quality of TGV wet etching directly governs subsequent metallization filling, electrical performance, and thermo-mechanical reliability. To prevent evaluating process efficacy through a single parameter, this paper synthesizes TGV wet-etching performance into five key evaluation metrics: aspect ratio, sidewall roughness, sidewall verticality, diameter uniformity, and defect control. Strong coupling exists among these metrics. For example, increasing the aspect ratio improves integration density but also aggravates internal etchant depletion, byproduct removal difficulty, and subsequent metal filling challenges. Similarly, lowering sidewall roughness reduces electrical loss and stress concentration, but typically demands further optimization of laser modification, internal etchant exchange, and post-processing treatments.

**Figure 2:**
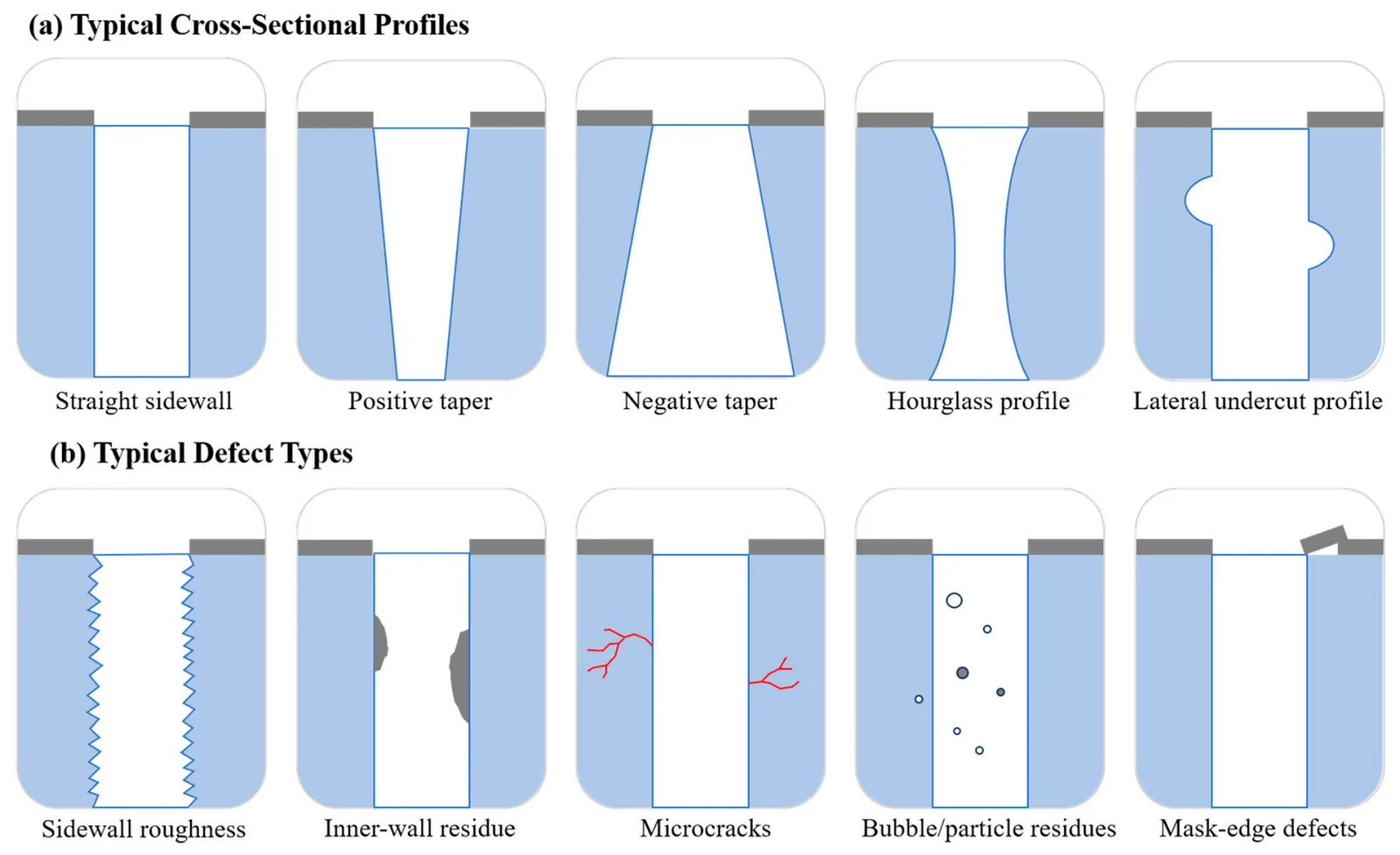
Schematic illustration of (**a**) typical TGV cross-sectional profiles and (**b**) representative defect types in wet etching.

To explicitly illustrate the specific morphological features corresponding to these evaluation metrics, Figure 2 categorizes common profile morphologies and defect types observed in TGV wet etching. The upper panel illustrates typical cross-sectional profiles including straight-walled, tapered, inverted-tapered, hourglass, and undercutting profiles, reflecting longitudinal etching capability, lateral etching suppression, and sidewall verticality. The lower panel outlines representative defects, such as sidewall roughness, internal residues, microcracks, bubble/particle entrapment, and mask-edge defects, demonstrating how surface quality, internal cleanliness, and defect control during etching impact subsequent metallization filling and packaging reliability.

As depicted in Figure 2, TGV morphology control is not merely about achieving greater etching depth, but rather necessitates striking a balance among profile shape, sidewall quality, internal residues, and defect mitigation. Consequently, subsequent evaluation should be conducted comprehensively across parameters including aspect ratio, sidewall roughness, sidewall verticality, diameter uniformity, and defect levels.

Aspect Ratio: The aspect ratio (AR) serves as a critical metric for evaluating the longitudinal processing capability and interconnect density of TGVs, defined as the ratio of via depth *H* to top-opening diameter *D*:

(9) $$\mathrm{AR}=\frac{H}{D}$$

where *H* is the via depth, and *D* is the top-opening diameter or equivalent diameter. Conventional HF-based etching typically exhibits isotropic characteristics, making it highly susceptible to undercutting—wherein the etchant laterally removes glass beneath the mask. This leads to via enlargement and pronounced sidewall tapering, thereby posing significant challenges in achieving high-AR structures [13,35]. In contrast, hybrid techniques such as laser-induced wet etching and selective laser etching can substantially elevate the AR by capitalizing on the etch-rate differential between modified and unmodified regions [18,20].

Sidewall Roughness: Sidewall roughness reflects the microscopic topography of the TGV sidewall, commonly quantified by the arithmetic average roughness *R_a_* or root-mean-square roughness *R_q_*:

(10) $$R_{a}=\frac{1}{L}\int_{0}^{L}\left|z(x)-\bar{z}\right|dx$$

(11) $$R_{q}=\sqrt{\frac{1}{L}\int_{0}^{L}{\left[z(x)-\bar{z}\right]}^{2}dx}$$

where *z*(*x*) represents the sidewall profile height, the average value of *z* is the mean profile height, and *L* is the sampling length. Research by Fang et al. demonstrated that by optimizing the LIWE process, the TGV sidewall roughness could be reduced from 1.257 µm to 25 nm, substantially improving microwave transmission performance [19].

Anisotropy and Verticality: Anisotropy describes the disparity between longitudinal and lateral etch rates, represented by the anisotropy factor *A*:

(12) $$A=1-\frac{v_{⊥}}{v_{v}}$$

where $v_{v}$ is the vertical etch rate, and $v_{⊥}$ is the horizontal etch rate. *A* value of *A* = 0 denotes nearly isotropic etching; as *A* approaches 1, lateral etching is effectively suppressed, yielding a profile closer to a vertical straight-walled structure. Sidewall verticality is typically evaluated via cross-sectional scanning electron microscopy (SEM) imaging to assess sidewall taper and its compatibility with subsequent metallization.

Diameter Uniformity: Diameter uniformity measures the processing consistency across large-area or high-density arrays, commonly represented by the relative standard deviation (RSD):

(13) $$\mathrm{RSD}=\frac{\sigma_{D}}{\bar{D}}\times 100\%$$

where $\sigma_{D}$ is the standard deviation of via diameters, and the average value of *D* is the average via diameter. For panel-level or wafer-level TGV arrays, a smaller RSD indicates superior process stability. Hou et al. achieved high-consistency processing for large-scale TGV arrays by incorporating ultrasonic assistance, temperature control, and substrate motion, achieving a via diameter RSD of less than 1% [36].

Geometric Morphology and Defect Levels: Beyond quantitative parameters, features such as TGV cross-sectional profiles, diameter consistency along the depth direction, internal residues, microcracks, bubble replication, particle contamination, and mask-edge defects profoundly influence process evaluation. For high-frequency interconnections and high-reliability packaging, an ideal morphology requires not only high depth, but also simultaneous fulfillment of smooth sidewalls, uniform via diameters, minimal defects, and ease of metal filling.

To establish a clearer relationship among process parameters, etching morphology, and subsequent performance, Table 1 summarizes the primary evaluation metrics in TGV wet etching, their characterization details, and their downstream impacts on subsequent processes and device performance.

The aspect ratio is defined as the ratio between via depth and diameter, while sidewall roughness, sidewall verticality, diameter uniformity, and etching defects are used to describe the surface characteristics, geometric variations, and structural imperfections generated during wet etching.

As indicated in Table 1, the quality evaluation of TGV wet etching should comprehensively encompass multiple dimensions, including geometric formation, surface quality, array consistency, and defect control. Among these, aspect ratio and sidewall verticality primarily reflect the longitudinal via-formation capability and lateral etching suppression; sidewall roughness and defect levels directly govern seed-layer coverage, electroplating fill integrity, and high-frequency signal loss; while diameter uniformity dictates the stability and yield of large-area array processing. Consequently, the evaluation of TGV wet etching must not rely solely on etch depth or single-via morphology, but rather require a holistic assessment integrated with downstream metal filling, electrical performance, and packaging reliability.

### 2.4. Summary of Issues and Transition to Technological Routes

The preceding sections have systematically summarized the fundamental question of “Why is TGV wet etching challenging?” from a mechanistic perspective. HF-based etching exhibits high etching efficiency, serving as the predominant chemical system in current glass TGV processing. Conversely, alkaline etching offers certain advantages in mask compatibility and surface quality, though its etch rate and high-aspect-ratio processing capabilities remain constrained. For deep-via structures, surface reaction kinetics, etchant diffusion, reaction byproduct removal, and the amorphous nature of glass jointly dictate etching morphology, rendering conventional wet etching highly susceptible to undercutting and pronounced sidewall tapering. These intrinsic limitations indicate that improving TGV fabrication requires coordinated optimization of etching chemistry, mass transport, and morphology control rather than simply increasing the etching rate.

Therefore, the core objective of subsequent technological routes is not simply elevating the etch rate, but rather achieving comprehensive optimization focused on aspect ratio, sidewall roughness, sidewall verticality, diameter uniformity, and defect control. Based on the aforementioned mechanisms and evaluation metrics, the following sections will further analyze specific developmental pathways, including conventional HF/BHF wet etching, material composition tailoring, mask and flow-field assistance, laser-induced/selective laser modification, and multiphysics assistance. The relationship between morphology quality and electrical performance will also be discussed to clarify the practical requirements for reliable TGV applications.

## 3. Overview of TGV Wet-Etching Technological Routes

Based on the synthesis of etching mechanisms and evaluation metrics presented in the preceding sections, the subsequent review adopts a problem–method–effect technological framework rather than a chronological listing of literature. Specifically, the fundamental roles and morphological limitations of conventional HF/BHF wet etching are analyzed first, followed by separate discussions on material composition tailoring, mask and flow-field assistance, laser-induced/selective laser modification, multiphysics assistance, and the coupling between surface quality and electrical performance.

### 3.1. Conventional HF/BHF Wet Etching: Process Fundamentals and Morphological Limitations

Conventional wet etching using HF or buffered HF (BHF) represents one of the most mature and cost-effective processing routes in glass micromachining. This approach typically employs etching systems such as HF, HF/HCl, or HF/$NH_{4}F$ solutions in conjunction with mask materials like Cr/Au, Cr/photoresist, SiC, or thick photoresist to achieve pattern transfer onto glass surfaces. Owing to its relatively simple workflow and high etching throughput, conventional wet etching retains substantial practical value for low-aspect-ratio micro-grooves, micro-cavities, and preliminary via processing [15,35]. Williams and Muller systematically tabulated the etch rates of common microfabrication materials early on, underscoring that etch rate, material selectivity, and mask compatibility constitute foundational parameters in wet-micromachining process design [16]. These advantages make HF/BHF-based etching a benchmark process for evaluating subsequent morphology-control strategies, despite its inherent limitations in achieving high-aspect-ratio TGV structures.

However, the fundamental limitation of conventional HF/BHF wet etching resides in its isotropic character, which stems directly from the amorphous nature of glass. The etchant removes material not only along the via-depth direction, but also laterally beneath the masking layer, causing via enlargement and pronounced sidewall tapering. Ding et al. extended mask lifetime in 49% HF using AZ4620-reinforced Cr/Au masks, enabling deep etching of 500-μm-thick Borofloat 33 glass; nevertheless, significant undercutting persisted, yielding sidewall taper angles of approximately 45°–50° [14]. This demonstrates that mask reinforcement merely delays process failure rather than fundamentally breaking through isotropic constraints.

Overall, the contribution of conventional HF/BHF wet etching lies in offering a mature, low-cost baseline processing platform. Its primary drawbacks, however, manifest as limitations in achieving high aspect ratios, vertical sidewalls, and precise dimensional control. Consequently, this route is better suited for low-aspect-ratio geometries or as a pre-/post-processing step within hybrid workflows, rather than independently fulfilling the strict demands of high-performance TGVs for high AR and vertical straight-walled profiles.

### 3.2. Material Composition-Tailored Etching: Inducing Rate Differentials via Glass Formulation

To break through the constraints of ideal isotropic etching, some studies approach the problem from the perspective of glass materials themselves, utilizing differences in reactivity, dissolution products, and passivation behaviors among various glass components in HF-based systems to construct quasi-anisotropic etching conditions. Manasa et al. compared the etching behavior of soda-lime glass with that of Borofloat-33 glass in HF/BHF systems, finding that soda-lime glass—containing higher concentrations of network modifiers such as $Na^{+}$ and $Ca^{2+}$—dissolves in a manner closely approaching isotropic behavior. Conversely, the higher $B_{2}O_{3}$ content in Borofloat-33 glass alters localized reaction products and surface passivation behavior, causing it to exhibit non-isotropic etching characteristics [37].

Mechanistically, material composition tailoring does not restrict lateral etching directly through external equipment; rather, it indirectly alters the differential between longitudinal and lateral etch rates by modifying the glass network structure, non-bridging oxygen concentration, soluble/insoluble reaction product formation, and localized passivation layer development. The primary advantage of this approach lies in improving etching morphology from the material design level; however, its efficacy remains heavily dependent on glass formulation and network structure, rendering the process window difficult to translate directly across different commercial glass substrates.

Consequently, material composition tailoring serves as a solid theoretical foundation for understanding etch rate disparities and selecting substrate materials, as well as guiding process development for specific borosilicate or photosensitive glasses. Nevertheless, for universal TGV manufacturing, relying solely on compositional variations remains insufficient to reliably achieve high-aspect-ratio structures with vertical sidewalls.

The influence of glass composition can be further understood by comparing representative glass systems used in TGV and related glass micromachining studies. Soda-lime glass tends to exhibit nearly isotropic dissolution in HF-based solutions, whereas the multicomponent network of borosilicate glass can alter local dissolution and passivation behavior. Fused silica provides a relatively uniform SiO_2_-dominated network and is widely used in laser-assisted selective etching, where high etch-rate selectivity can be introduced between modified and unmodified regions. Photosensitive glass, in contrast, relies on exposure and thermal treatment to create selectively etchable regions. These differences indicate that glass composition affects not only the intrinsic dissolution behavior but also the applicable morphology-control mechanism and achievable process window. A comparison of representative glass systems is summarized in Table 2.

### 3.3. Masking and Flow-Field Assisted Etching: Suppressing Undercutting via External Confinement

Beyond material factors, masking systems and flow-field confinement represent another crucial pathway for refining wet-etching morphology. Unlike material composition tailoring, this strategy focuses on restricting undesired lateral etchant transport through external confinement rather than modifying the intrinsic dissolution behavior of glass. The core issue with traditional solid-state masks includes stress cracking, pinholes, hydrophilic defects, and mask-edge seepage. These defects allow localized etchant intrusion and trigger unintended etching in non-designed regions. Existing studies indicate that employing multilayer metal/photoresist composite masks, low-stress mask materials, and hard-baked photoresists can enhance mask corrosion resistance, yet fully eliminating undercutting and mask-edge defects remains challenging [14,15].

To address these limitations, Mu et al. proposed the Laminar Flow Assisted Wet Etching (LAWE) method, transforming conventional full-immersion etching into a confined-flow process. This technique utilizes a stable laminar flow to establish a “liquid mask,” confining the HF etchant contact strictly to targeted areas while protective fluids flanking both sides restrict lateral HF diffusion, thereby suppressing lateral etching. Experimentally, LAWE elevated the aspect ratio of glass microchannels to approximately 1, demonstrating the potential of flow confinement to overcome the geometric limitation imposed by conventional isotropic wet etching [39].

The contribution of this technological route lies in demonstrating that external flow-field confinement can enhance etching directionality without altering the intrinsic glass material. However, its limitations are equally prominent, including microfluidic system complexity, restrictions on processing dimensions and regions, and insufficient compatibility with large-area, high-density TGV arrays. Thus, masking and flow-field assisted etching are currently more suitable for microchannels, localized pattern processing, or hybrid processes where precise local confinement is required, while their application to large-area high-density TGV fabrication remains challenging.

### 3.4. Laser-Induced and Selective Laser-Modified Wet Etching: Critical Breakthroughs for High-Aspect-Ratio TGVs

**Figure 3:**
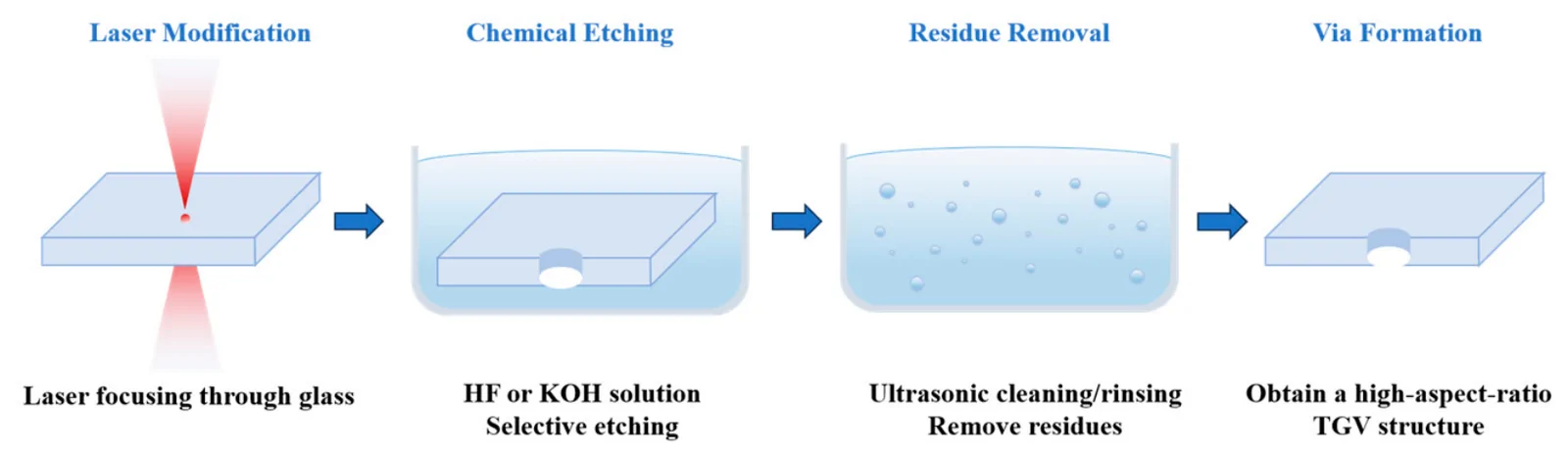
Schematic of the fabrication process of TGV by laser-modified assisted selective wet etching.

Laser-induced and selective laser-modified wet etching have emerged in recent years as core methodologies for achieving high-aspect-ratio TGVs. The underlying concept involves utilizing laser irradiation to form highly reactive modified regions, nanochannels, or localized defect structures within the bulk or on the surface of glass, followed by selective removal of these modified zones via wet etching. Unlike conventional wet etching, the directionality of this approach derives primarily from the etch-rate differential between modified and unmodified regions, rather than relying on intrinsic crystallographic selectivity. To intuitively illustrate the fundamental process of this hybrid technology, Figure 3 outlines a typical process flow for fabricating TGVs via laser-modification-assisted selective wet etching.

As shown in Figure 3, this process generally encompasses four stages: laser modification, chemical selective etching, residue removal, and via formation. The laser first induces localized modified regions within the bulk or on the surface of the glass, endowing these zones with elevated dissolution rates in etchants such as HF or KOH. Subsequently, the etchant preferentially removes the modified regions, and residues are evacuated through cleaning or rinsing, ultimately giving rise to the TGV structure. Compared to conventional wet etching, the key advantage of this strategy lies in establishing directional selectivity through the etch-rate differential between modified and unmodified regions, thereby boosting longitudinal processing capability while suppressing lateral etching.

Under this technological framework, variations among approaches like LIBWE, LIWE, SLE, and LIDE reside primarily in laser interaction zones, modification mechanisms, etchant chemical systems, and downstream morphology control strategies. The following discussion incorporates representative studies to further analyze the progress of these techniques in high-aspect-ratio formation, sidewall quality control, and processing throughput enhancement. Marcinkevičius et al. demonstrated early on that femtosecond lasers could induce localized modification within fused silica, enabling 3-D microstructure fabrication through subsequent chemical etching [40]; Juodkazis et al. further established the feasibility of 3-D microfabrication in polymers and glasses using femtosecond pulses [41]. Subsequently, Bellouard et al. utilized femtosecond lasers and chemical etching to fabricate high-aspect-ratio microfluidic channels and tunnel structures, while Hnatovsky et al. further applied focused femtosecond laser irradiation and selective chemical etching to prepare glass microchannels, highlighting the distinct advantages of this route for deep-structure microfabrication within transparent glasses [42,43].

LIBWE generates localized thermal effects, shockwaves, and bubble dynamics at the interface between transparent glass and an absorbing liquid, enabling high-precision material removal. Kawaguchi et al. fabricated microgrooves with a width of approximately 7 μm and a depth of roughly 420 μm on quartz glass using LIBWE, achieving an aspect ratio of nearly 60 and demonstrating a longitudinal processing capability far surpassing that of conventional wet etching [18]. Kwon et al. further optimized the LIBWE process from a scanning path perspective, mitigating morphological errors and bubble accumulation effects via error-compensation scanning, which underscores that laser path design is equally vital for processing uniformity and surface roughness [44].

With respect to sidewall verticality in TGVs, additive-assisted laser-induced wet etching provides new optimization avenues. Zhang et al. introduced poly(diallyldimethylammonium chloride) (PDDA) additives into an HF-based system to form an etch-inhibition layer on the glass surface, thereby suppressing the lateral etch rate. This enabled TGVs with an aspect ratio of 23:1 to achieve a verticality evaluation value of approximately 0.77, significantly refining via sidewall morphology [45]. This result demonstrates that additives can adjust the reaction environment at the etching interface and synergize with the selective dissolution of laser-modified zones to collaboratively improve etching directionality and sidewall quality. Similarly, Dou et al. investigated the impacts of additives such as isopropyl alcohol (IPA), acetic acid, and sodium dodecyl sulfate (SDS) on BOE etching of Z-cut quartz, discovering that appropriate additive concentrations regulate etch rates and post-etch surface conditions; specifically, SDS significantly reduced post-etch surface roughness, proving that additive assistance serves as a key approach for refining wet-etched surface quality on glass and quartz [46].

**Figure 4:**
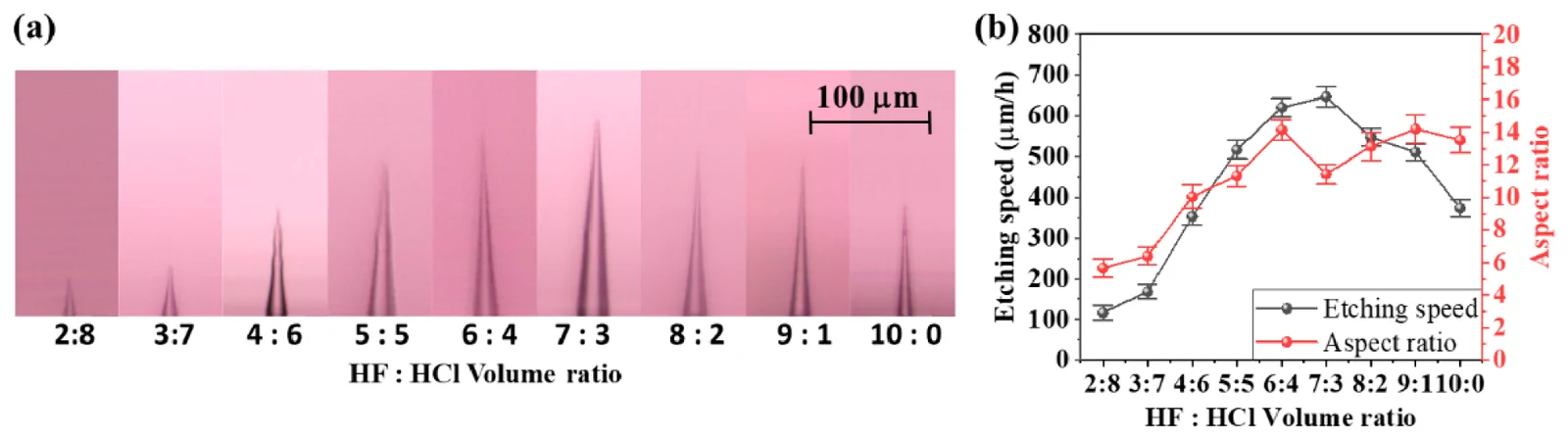
Influence of HF:HCl volume ratio on laser-induced deep etching of NXT glass: (**a**) optical microscope images of etched samples obtained at different HF:HCl volume ratios; (**b**) corresponding variations in etching speed and aspect ratio. Reproduced with permission from Bang, S.; Asghar, G.; Hwang, J.; Lee, K.S.; Jung, W.; Mishchuk, K.; Kim, H.; Lee, K.G., *Opt. Express*; published by Optica Publishing Group, 2025 [21].

Building upon these foundations, Liu et al. combined femtosecond laser-induced nanochannels with selective wet etching to fabricate fused silica TGVs with aspect ratios ranging from 11.7 to 61.3, while boosting the processing throughput to approximately 10,000 TGVs/min, revealing strong industrial potential for co-optimizing high aspect ratio and high throughput [20]. In a similar vein, Bang et al. systematically investigated the LIDE process for NXT glass and demonstrated that both laser modification parameters and subsequent chemical etching conditions strongly affect the final etching response [21]. As shown in Figure 4, variations in the HF:HCl volume ratio produce clear differences in the etched microhole morphology, while simultaneously affecting the etching speed and achievable aspect ratio. These results provide a direct process–morphology comparison and demonstrate that the chemical composition of the etchant is a critical parameter for balancing etching efficiency and high-aspect-ratio structure formation. Although the investigated structures were not specifically designed for packaging TGV applications, they still provide valuable process-related evidence for understanding morphology control during laser-assisted wet etching.

**Figure 5:**
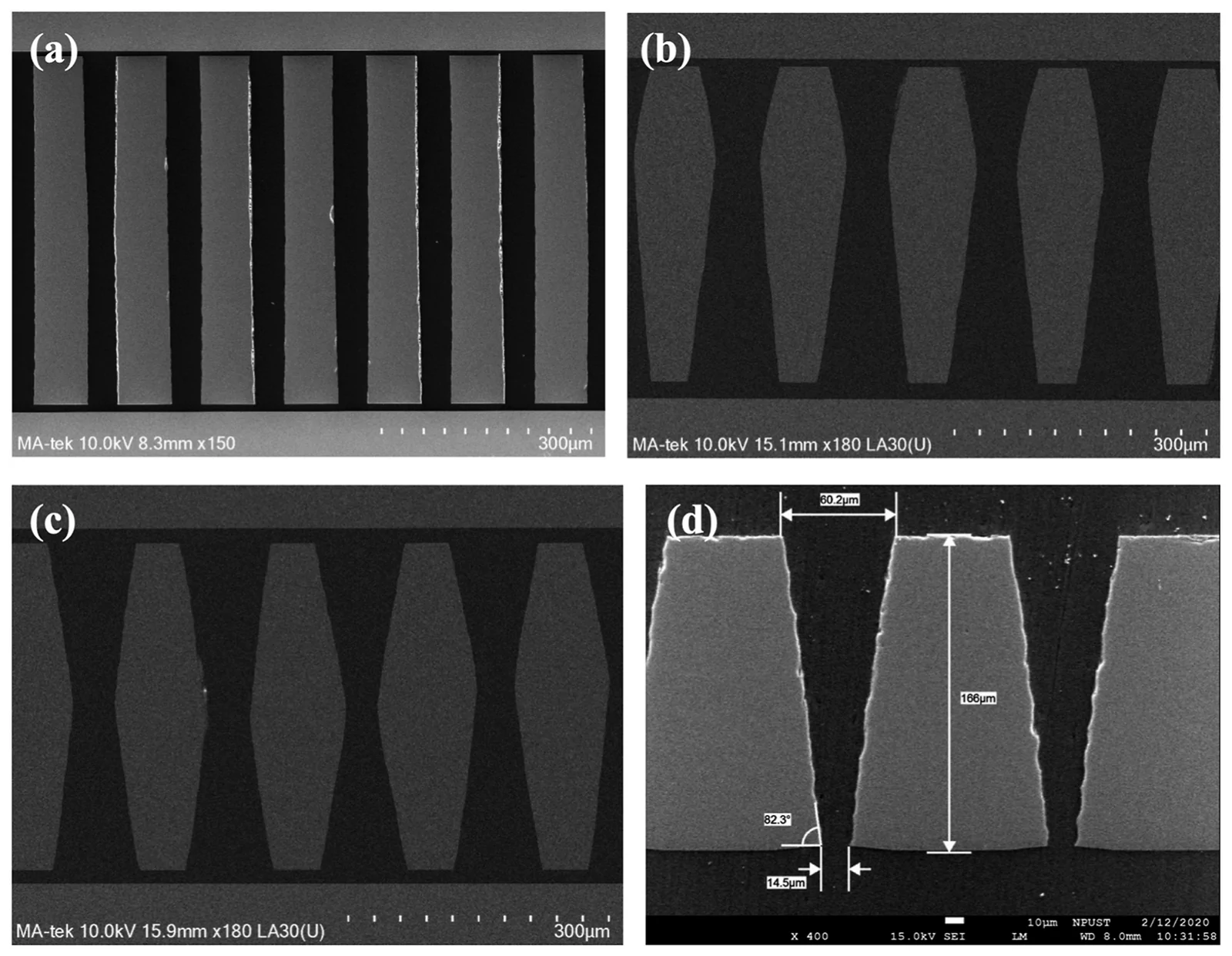
Representative TGV cross-sectional morphologies with different shapes and taper angles: (**a**) straight; (**b**,**c**) hourglass-shaped with different waist heights; and (**d**) isosceles. Reproduced with permission from Lee, M.-K.; Yu, J.-Z.; Chang, H.-Y.; Chang, C.-Y.; Liu, C.-S.; Lin, P.-C., *AIP Adv*, published by AIP Publishing, 2022 [47].

Beyond chemical etching optimization, laser modification also provides substantial flexibility in tailoring the final TGV profile. Lee et al. demonstrated that variations in laser focusing position, laser power, and subsequent wet-etching conditions could generate straight, hourglass-shaped, and tapered TGV profiles, as shown in Figure 5 [47]. These experimental results further demonstrate that the final TGV morphology is determined not only by successful via formation, but also by the spatial distribution of laser modification and its interaction with subsequent chemical etching.

Beyond the control of aspect ratio and via profile, another developmental frontier for selective laser etching is improving processing speed, complex-geometry capability, and morphological predictability. Hermans et al. investigated high-speed selective laser-induced etching of fused silica in KOH systems, while Gottmann et al. demonstrated expanded manufacturing capabilities for complex 3-D precision quartz glass structures in terms of complexity and speed enhancement [48,49]. In recent years, research on polarization-insensitive selective etching, deep-layer aberration suppression, and 3-D precision manufacturing of ultra-low expansion (ULE) glass has further shown that laser-assisted wet etching is expanding from single-via drilling to high-precision, multifunctional glass microstructure fabrication [50,51,52]. This provides a crucial technological baseline for high-aspect-ratio TGV formation, sidewall quality refinement, and cross-substrate consistency enhancement.

Overall, the greatest advantage of laser-induced and selective laser-modified wet etching lies in achieving high aspect ratios and vertical sidewalls while maintaining low roughness and high processing precision. However, its drawbacks stem from high equipment capital expenditure and complex process windows that are hypersensitive to laser parameters, focal positioning, modification uniformity, and downstream etching conditions. Future research must address issues regarding high-throughput manufacturing, cross-substrate consistency, and in-line process monitoring.

### 3.5. Multiphysics and Process Monitoring Assistance: Toward Array Uniformity and Closed-Loop Control

For panel-level or wafer-level TGV arrays, achieving a high aspect ratio in an isolated single via is insufficient; as the via density increases, process non-uniformity caused by local mass-transfer limitations and reaction-product accumulation becomes increasingly critical. Consistent performance across thousands to tens of thousands of TGVs in terms of diameter, depth, sidewall morphology, and defect levels is imperative. Multiphysics-assisted etching primarily utilizes ultrasonics, temperature control, substrate movement, and fluid agitation to promote internal etchant replenishment and accelerate the expulsion of gas bubbles and reaction byproducts, thereby reducing inter-via etching disparities across the array. Hou et al. constructed a wet-etching system integrating ultrasonic assistance, temperature control, and substrate motion, achieving consistent processing of over 26,000 TGVs on Corning Eagle XG glass, with the via diameter uniformity metric reduced below 1% [36].

The photosensitive glass route relies on an “exposure–thermal treatment–selective wet etching” sequence to yield micro-via structures with high selectivity. Yook et al. utilized photosensitive glass to fabricate TGVs and embedded spiral inductors, obtaining via structures with diameters of approximately 20–60 μm and aspect ratios of roughly 4:1, further demonstrating the application value of TGVs in integrated passive devices [38]. The advantage of this route lies in superior process selectivity and high sidewall quality; however, it generally depends on specific photosensitive glass formulations, which constrains material selection and inflates costs.

In addition, process monitoring and parameter optimization are emerging as critical directions for enhancing TGV manufacturing stability. Kim et al. established a depth-estimation method based on LIBWE acoustic signals and wavelet transforms, designing a closed-loop control system that maintained microchannel processing errors at a minimal level [53]. Ouyang et al. introduced deep learning and Bayesian optimization into TGV laser drilling parameter tuning, achieving automated SEM image-based quality evaluation and iterative parameter optimization [54]. These developments indicate that TGV manufacturing is progressively transitioning from empirical trial-and-error approaches toward data-driven, closed-loop control systems.

### 3.6. Impact of Etch Morphology on Metal Filling and Electrical Performance

TGV etching morphology should ultimately be evaluated through downstream metallization quality, electrical performance, and device reliability. Galler et al. investigated MMIC-to-dielectric waveguide transitions in glass packaging above 150 GHz, demonstrating that glass-based packaging imposes stringent demands on geometric precision, dielectric loss, and interconnect integrity in millimeter-wave and high-frequency interconnect scenarios [13]. Excessive sidewall roughness leads to discontinuous seed-layer coverage, non-uniform electroplated thickness, localized electric field concentration, and interfacial stress concentrations, thereby inducing voids, seams, signal loss, and thermo-mechanical failures. Consequently, surface quality should not be viewed merely as a morphological parameter, but rather as a pivotal intermediate variable bridging the etching process to ultimate device performance.

**Figure 6:**
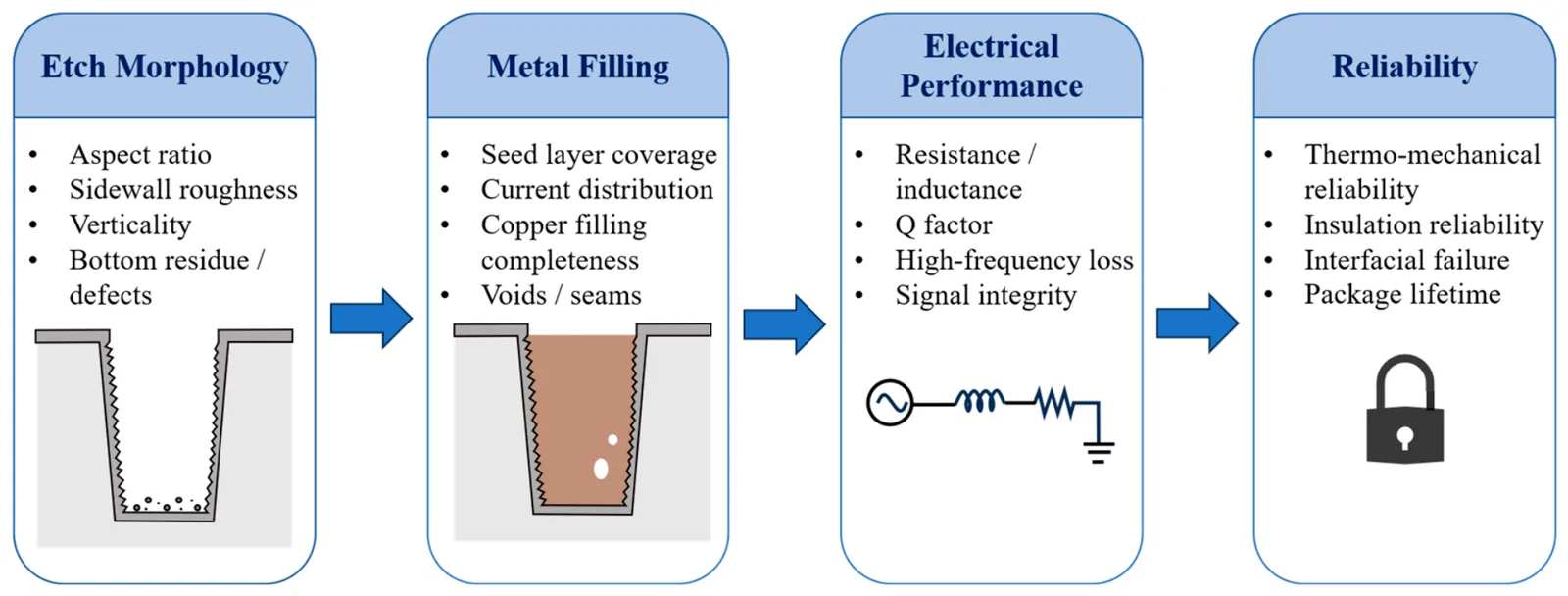
Schematic of the influence mechanism of TGV etch morphology on metal filling, electrical performance, and reliability.

To further clarify the cascading impact of etching morphology on downstream processes and device performance, Figure 6 illustrates the chain of influence encompassing “etch morphology–metal filling–electrical performance–reliability.” The schematic highlights how key morphological features, including aspect ratio, sidewall roughness, verticality, internal residues, and diameter uniformity, influence seed-layer coverage, current distribution, and copper filling quality. Subsequently, filling defects and interfacial quality alter electrical attributes such as resistance, inductance, Q-factor, high-frequency attenuation, and signal integrity, ultimately governing thermo-mechanical reliability, dielectric reliability, and packaging operational lifespan.

Grounded in the operational relationships depicted in Figure 6, the evaluation of TGV wet etching must extend beyond mere geometric formation to encompass the direct influence of morphological parameters on metallization and electrical behavior. An elevated aspect ratio intensifies the challenge of achieving continuous seed-layer coverage and defect-free superconformal copper electrodeposition; excessive sidewall roughness triggers localized electric field concentration, interfacial stress accumulation, and heightened high-frequency attenuation; while internal residues or filling defects compromise dielectric and thermo-mechanical reliability. Thus, etching morphology functions as a critical intermediate variable linking front-end wet-etching processes to downstream device performance.

Empirical studies further validate this morphology-performance coupling. Grosse et al. utilized backside TGVs to realize electrical interconnections for sensing structures within a bidirectional 3-omega thermal characterization platform, demonstrating that TGV structural quality dictates the application stability of functional device platforms [55]. For high-aspect-ratio or complex sidewall topographies, continuous coverage and adhesion quality of the seed/barrier layer are especially crucial; investigations by Peters et al. on electroless metallization over complex surfaces revealed that seed-layer morphology, adhesion strength, and resistivity significantly govern subsequent deposition quality and high-frequency signal loss [56]. Fang et al. systematically evaluated the influence of TGV sidewall roughness on microwave transmission characteristics, reducing sidewall roughness from 1.257 μm to 25 nm via LIWE process optimization and developing a resistance–inductance–conductance–capacitance (RLGC) model incorporating sidewall roughness effects, thereby proving that elevated roughness increases equivalent resistance, inductance, and dielectric loss [19]. Wang et al. further compared the performance of embedded 3-D inductors across distinct TGV topographies, demonstrating that smooth hyperbolic-sidewall TGVs obtained by optimizing laser paths and wet-etching channels substantially enhance inductance density and quality factor Q [57].

Taken together, these studies indicate that different morphological parameters influence distinct stages of downstream integration: sidewall roughness primarily affects high-frequency electrical performance, sidewall inclination governs local copper deposition behavior, and the overall via profile influences passive-device performance. Therefore, TGV wet etching should be evaluated through the coupled relationship among morphology, metallization, and electrical performance rather than by geometric quality alone.

### 3.7. Comparison of Main Technological Routes and Representative Performance

To enable a more systematic comparison of different morphology-control strategies, Table 3 summarizes representative studies according to glass type, structural configuration, etchant or assistance method, key reported metrics, and the resulting morphology-control outcomes. Because some studies focus directly on TGV fabrication whereas others investigate related glass microstructures, the evidence type is explicitly distinguished in the table. The latter studies are included to clarify transferable process–morphology relationships rather than to serve as direct TGV performance benchmarks.

As summarized in Table 3, different technological routes target different aspects of morphology control. Conventional HF-based wet etching remains attractive because of its process maturity and low cost, but is strongly constrained by isotropic dissolution, undercutting, and sidewall tapering. Flow-field-assisted approaches mainly improve lateral-etch suppression, whereas laser-induced and selective wet etching provide greater flexibility in controlling aspect ratio, sidewall profile, and etching selectivity. Multiphysics-assisted processing, in contrast, primarily contributes to large-area array consistency and manufacturing scalability. Because the reported results were obtained using different glass compositions, structural dimensions, etchants, and assistance conditions, the numerical values should not be interpreted as strictly equivalent cross-study benchmarks. Instead, the comparison is intended to identify process-dependent trends and the relative strengths and limitations of different morphology-control strategies.

## 4. Challenges and Future Outlook

TGV wet-etching technology has progressed from traditional isotropic HF/BHF etching to a diversified technological landscape encompassing material composition tailoring, mask and flow-field confinement, laser-induced modification, multiphysics assistance, and data-driven optimization. Shifting from the preceding focus on etching mechanisms, morphological metrics, and technological routes, this section further addresses the critical bottlenecks remaining from an industrial application perspective. Overall, the current challenge is no longer merely “whether a through-via can be etched,” but rather whether TGV structures with high aspect ratios, low roughness, vertical sidewalls, low defect levels, and good metallization compatibility can be reliably produced over large-area glass substrates. These future research directions arise directly from the limitations identified in the preceding technological routes. Conventional HF/BHF etching is constrained by isotropic dissolution and undercutting, while laser-assisted and multiphysics-assisted approaches improve morphology control but introduce greater process sensitivity and system complexity. Therefore, future development should focus on balancing aspect ratio, throughput, array uniformity, metallization compatibility, process stability, and environmental safety rather than optimizing a single morphology metric.

**Figure 7:**
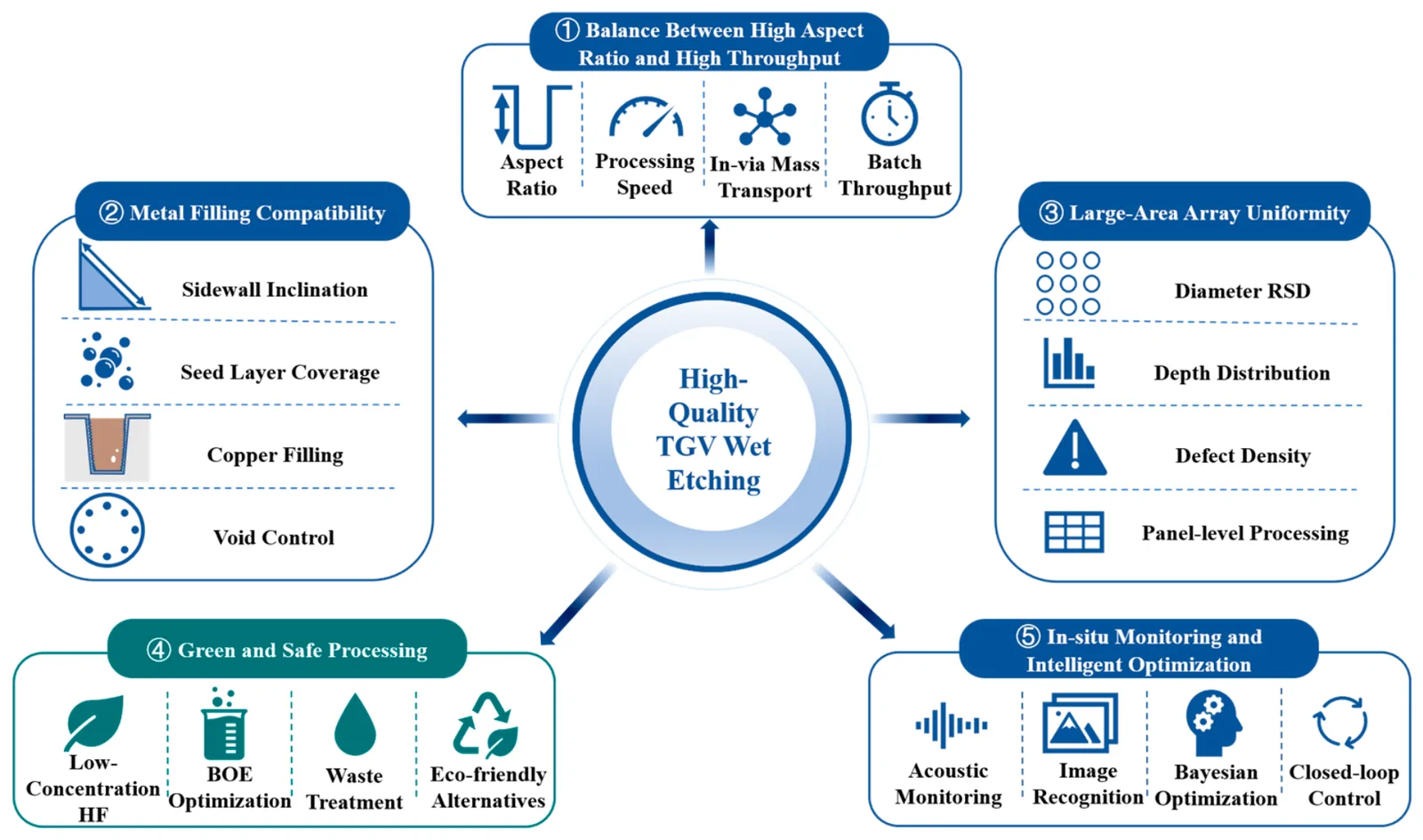
Roadmap of future development for high-performance glass-based TGV wet etching for packaging applications.

To outline future development directions for high-performance TGV wet etching in packaging applications, Figure 7 centers on “High-Quality TGV Wet Etching” and synthesizes subsequent research priorities into five thrusts: balancing high aspect ratio with high-throughput manufacturing, metal filling compatibility, large-area array uniformity, green and safe processes, and in situ monitoring with intelligent optimization. Among these, balancing high aspect ratios with high-throughput manufacturing primarily addresses etching efficiency, internal etchant replenishment, and reaction byproduct evacuation; metal filling compatibility emphasizes the coupling among via sidewall morphology, seed-layer coverage, and superconformal copper electrodeposition quality; large-area array uniformity focuses on via diameter RSD, depth distribution, and defect density; while green and safe processes, together with in situ monitoring and intelligent optimization, target improvements in operational safety, environmental friendliness, and process stability.

As illustrated in Figure 7, the future evolution of TGV wet etching exhibits pronounced characteristics of system-level synergy. Front-end etching processes must not only yield high aspect ratios and low surface roughness, but must also simultaneously accommodate throughput efficiency, array uniformity, metal filling compatibility, and process safety. Grounded in this logic, the following subsections detail discussions covering high aspect ratio versus high throughput, metal filling and reliability, large-area array defect control, green and safe processes, as well as in situ monitoring and intelligent optimization.

### 4.1. Primary Technical Challenges Currently Faced

#### 4.1.1. Balancing Extreme Aspect Ratio and High-Throughput Manufacturing

High aspect ratio represents a core target continuously pursued in TGV wet-etching technology. Hybrid routes such as LIBWE have demonstrated the capability to fabricate glass microstructures with aspect ratios exceeding 60 [18]. Liu et al. combined selective laser modification with wet etching to fabricate TGVs in fused silica with aspect ratios ranging from 11.7 to 61.3, while boosting the laser processing speed to approximately 10,000 TGVs/min [20]. Furthermore, Bang et al. investigated the LIDE process on NXT glass, proving that the synergistic optimization of laser parameters, HF/HCl chemical ratios, and ultrasonic assistance significantly accelerates etch rates and elevates aspect ratios [21].

However, achieving an extreme aspect ratio is not synonymous with industrial manufacturing capability. Practical manufacturing demands simultaneous consideration of point-by-point laser processing time, modification uniformity, downstream wet-etching process windows, internal byproduct evacuation, and batch equipment tact time. Solely pursuing higher ARs may induce via diameter fluctuations, increased sidewall defect density, or heightened metallization challenges. Consequently, future process evaluations must transition from single-point extreme metrics toward a holistic balance among “aspect ratio–throughput–defect density–downstream filling compatibility.”

#### 4.1.2. Metal Filling and Thermo-Mechanical Reliability of High-Aspect-Ratio TGVs

As aspect ratios escalate, TGV metallization, particularly electroplated copper filling, becomes increasingly challenging. Deep high-aspect-ratio geometries aggravate non-uniformities in internal additive transport, current distribution, and copper deposition front propagation, thereby increasing the risk of voids, seams, and localized over-plating. Drawing from 3-D interconnect manufacturing experience, high-aspect-ratio copper filling typically relies on the synergistic interplay of additives, including suppressors, accelerators, levelers, and chloride ions, to establish a bottom-up superconformal copper electrodeposition process. Research by Moffat et al. on superconformal copper electrodeposition revealed that localized additive coverage, curvature variations within micro-vias or trenches, and electrolyte distributions of metal ions and additives dictate the propagation and morphological evolution of the copper deposition interface, ultimately determining the formation of void or seam defects [58].

For TGV structures, front-end etching morphology directly dictates downstream metallization quality. Sidewall taper angles, sidewall roughness, and internal residues govern not only seed-layer coverage and barrier-layer continuity, but also alter localized current distribution during electrochemical deposition. Relevant electrochemical simulation studies demonstrate that excessive sidewall taper angles or improper suppressor concentrations disrupt localized adsorption and copper deposition profiles, leading to over-passivation, X-shaped voids, or incomplete filling [27]. Therefore, the metal filling issue in high-aspect-ratio TGVs cannot be solved in isolation during the electroplating stage; rather, it demands strict control over sidewall taper angles, sidewall roughness, internal residues, and via diameter consistency along the substrate thickness starting from the etching phase.

In addition to filling defects, the thermal expansion coefficient mismatch between copper and glass introduces thermo-mechanical reliability risks. Lai et al. synthesized stress concentration and interfacial failure modes of copper-filled TGV structures under thermal cycling, highlighting that high-aspect-ratio structures are particularly prone to thermal stress accumulation during electroplating, annealing, and operational service [26]. Consequently, future high-aspect-ratio TGV fabrication requires establishing a synergistic design framework connecting “etching morphology–seed-layer coverage–copper filling–thermo-mechanical reliability,” balancing filling integrity and long-term reliability alongside the pursuit of high ARs.

#### 4.1.3. Large-Area Array Uniformity and Defect Control

For wafer-level or panel-level applications, TGV manufacturing demands the simultaneous creation of thousands to tens of thousands of through-vias across large-area glass substrates. Under these conditions, localized thermal fluctuations, etchant concentration variations, non-uniform flow fields, bubble adsorption, particulate contamination, and mask edge defects become magnified, resulting in inter-via disparities in diameter, depth, sidewall angle, and defect density across the array. Hou et al. achieved consistent processing of over 26,000 TGVs via ultrasonic assistance, temperature control, and substrate motion, reducing the surface via diameter c-value to 0.73%, thereby underscoring the critical role of multiphysics assistance in elevating array uniformity [36].

From an industrial manufacturing perspective, however, via diameter uniformity alone does not fully represent etching quality. Defects such as bubble replication, microcracks, localized particulate residue, internal residues, and mask edge seepage similarly compromise downstream dielectric reliability, metallization integrity, and high-frequency performance. Consequently, future research must establish a multi-metric quality evaluation system for large-area arrays, unifying via diameter RSD, depth distribution, sidewall roughness, defect density, and electrical testing outcomes into process window determination.

#### 4.1.4. Development of Low-Cost and Environmentally Friendly Processes

HF-based systems remain among the most efficient and widely deployed chemical routes for glass wet etching. However, their extreme corrosiveness, toxicity, equipment degradation, and fluorine-containing wastewater treatment issues constrain green manufacturing and large-scale industrial adoption. Choi et al. systematically reviewed the environmental and safety challenges associated with HF-based systems in glass wet etching, evaluating potential alternatives such as alkaline etchants, buffered fluoride systems, and additive-assisted formulations [59]. Nevertheless, alkaline systems typically suffer from lower etch rates and limited adaptability across diverse glass compositions, making complete replacement of HF-based systems currently difficult.

It should be noted that while buffered oxide etchants (BOE) improve etch rate stability and process controllability, they intrinsically remain fluorine-containing wet-chemical systems. In their study on BOE deep wet etching of fused silica, Konstantinova et al. pointed out that mask corrosion resistance, etch rates, and profile isotropy remain primary limiting factors in deep-structure processing [17]. Therefore, the focus of future environmentally friendly processes lies not merely in searching for a single substitute etchant, but in implementing systematic solutions, including low-concentration fluorinated system optimization, closed-loop wastewater management, localized fluid delivery, dry-wet hybrid removal, and online safety monitoring.

### 4.2. Future Research Directions and Prospects

#### 4.2.1. Synergistic Optimization and Intelligence of Hybrid Processes

Future TGV wet-etching research must further transition from single-process parameter optimization toward the synergistic design of hybrid processes. Laser modification, additive assistance, flow-field confinement, and ultrasonic enhancement can individually govern selective dissolution within modified zones, lateral etch suppression, internal etchant replenishment, reaction byproduct evacuation, and sidewall quality refinement, respectively. The critical issue lies in deciphering the coupled interactions among these parameters rather than optimizing isolated variables in a vacuum.

Data-driven approaches offer a novel paradigm for resolving this challenge. Ouyang et al. applied deep learning and Bayesian optimization to automatically optimize TGV laser drilling parameters, achieving automated SEM image-based quality evaluation and iterative parameter optimization [54]. Coupled with the findings of Bang et al. regarding the synergistic optimization of laser, chemical, and mechanical parameters in LIDE [21], it becomes evident that future process development necessitates integrating machine learning-assisted multi-objective optimization frameworks to simultaneously constrain aspect ratio, sidewall verticality, roughness, defect density, and manufacturing throughput.

#### 4.2.2. Application-Oriented Matching of Glass Materials and Process Windows

Distinct glass formulations exhibit pronounced disparities in dielectric properties, coefficients of thermal expansion, elastic moduli, chemical stability, and etching byproducts. In their review of TGV technological advances, Seok and Jung emphasized that borosilicate glass, fused silica, and other low-loss glasses each offer specific application advantages, yet demand tailored process windows for via formation and metallization [10]. Consequently, future investigations should not merely compare “which glass demonstrates superior performance,” but rather establish a material-process co-selection framework linking “glass composition–laser modification response–wet-etching selectivity–metallization reliability.”

For high-frequency packaging applications, low dielectric loss and low surface roughness are paramount; conversely, for large-area panel-level packaging, dimensional stability, warpage mitigation, and cost efficiency take precedence. Simultaneously, TGV application domains are expanding beyond conventional packaging interconnects toward thermal characterization platforms, microsensing structures, and heterogeneous 3-D microsystems. Grosse et al. leveraged TGVs to construct a bidirectional 3-omega thermal characterization platform, demonstrating that TGVs can function as backside electrical interconnect structures in functional devices [55]. Meanwhile, Janssens et al. integrated glass TGVs with nanocrystalline diamond thin films to establish a diamond-glass platform tailored for 3-D micro/nano devices, microelectrodes, and quantum technologies [60]. Thus, establishing future TGV process windows must not rely solely on empirical parameters derived from a single glass formulation, but must encompass the synergistic relationships among glass composition, processing response, thin-film material compatibility, thermal expansion matching, and functional device application requirements.

Furthermore, materials such as ULE glass, fused silica, and low-loss packaging glasses exhibit variations in refractive index, optical absorption characteristics, and thermo-mechanical behavior, which further alter laser focusing, modification thresholds, and downstream etching selectivity [51,52]. Therefore, establishing future TGV process windows requires building transferable mapping relationships across diverse glass systems that connect “material response–laser modification–wet etching–morphological quality.”

#### 4.2.3. In Situ Monitoring and Closed-Loop Control

In situ monitoring represents a critical avenue for enhancing TGV manufacturing stability. Traditional etching processes frequently rely on post-process cross-sectional inspection, making it difficult to detect via depth deviations, bubble blockages, and localized over-etching in a timely manner. Kim et al. established a depth estimation method leveraging acoustic signals and wavelet transforms during the LIBWE process, designing a closed-loop control system that maintained microchannel fabrication errors at a remarkably low level [53]. Such investigations demonstrate that acoustic, optical, image-based, and electrochemical signals can all serve as effective information sources for online monitoring of the etching process.

Future work can further construct multi-sensor fusion closed-loop control systems that integrate real-time feedback on etching depth, via diameter variations, bubble dynamics, thermal fields, and etchant concentrations. Through closed-loop control systems and data-driven parameter optimization, variables such as laser scanning, ultrasonic power, fluid delivery flow rates, and etching duration can be dynamically adjusted. Only by embedding a “monitoring–identification–adjustment” loop directly into processing equipment can TGV wet etching transition from laboratory parameter optimization toward stable mass production.

#### 4.2.4. Standardized Testing and Reliability Database Construction

As TGV technology advances from laboratory research toward industrialization, establishing standardized testing protocols and unified data infrastructure is of paramount importance. Discrepancies persist across current literature regarding via diameter definitions, aspect ratio calculations, sidewall roughness sampling zones and characterization techniques, verticality evaluation methods, and defect criteria, rendering direct horizontal comparisons between studies difficult. Future efforts should establish a systematic evaluation methodology encompassing geometric morphology, electrical performance, metal filling quality, thermal cycling, high-temperature high-humidity (HTHH) endurance, and mechanical shock resistance.

Furthermore, a comprehensive TGV reliability database should be developed to compile glass substrate types, etching systems, laser parameters, via diameter/depth distributions, surface roughness, metal filling defect profiles, electrical loss metrics, and failure modes. This database will serve not only process traceability and failure analysis, but will also provide data support for machine learning modeling and design rule extraction. Related studies in IC packaging have also demonstrated that hybrid CNN–Transformer models can effectively identify packaging materials from images, further illustrating the potential of data-driven image analysis for automated packaging evaluation [61]. Compared with the TSV domain, TGV exhibits distinct characteristics in material systems, etching mechanisms, and high-frequency applications, underscoring an even greater need for dedicated evaluation standards tailored specifically for glass-based packaging.

## 5. Conclusions

Focusing on morphological control and process performance in TGV wet etching, this review provides a comprehensive analysis of the underlying mechanisms, evaluation metrics, technological routes, and future challenges. The analysis reveals that while conventional HF/BHF wet etching offers advantages in low cost and process maturity, it remains susceptible to lateral undercut, inadequate verticality, and limited aspect ratios—governed by the amorphous structure of glass, non-uniform internal etchant concentration profiles, and restricted evacuation of reaction byproducts. Tailoring material compositions, alongside mask and flow-field assistance, can improve etching directionality to a certain extent; however, their scope of applicability and scalability across larger area dimensions remain limited. Conversely, laser-induced/selective laser modification followed by wet etching, multiphysics assistance, and data-driven optimization offer highly promising pathways for fabricating TGVs characterized by high aspect ratios, low surface roughness, and superior uniformity.

Overall, the research trajectory of TGV wet etching is shifting from merely pursuing greater etching depths toward the holistic control of multiple morphological metrics including aspect ratio, sidewall roughness, sidewall verticality, via diameter consistency, and defect density, ultimately serving downstream metal filling, electrical performance, and reliability enhancement. Future research must undertake systematic optimizations centered on high-throughput manufacturing, cross-substrate uniformity, metal filling compatibility, green and safe processes, and closed-loop process control, thereby driving TGV wet etching from laboratory-scale high-performance structure fabrication toward stable industrial mass-production applications.

## Figures and Tables

**Table 1:** Definition, characterization methods, and influence of key morphology parameters in TGV wet etching.

Morphology Parameter	Definition	Characterization Methods	Influence on TGV Performance
Aspect ratio	Ratio between via depth and diameter	SEM, optical microscopy, WLI	Determines integration density and metal filling capability
Sidewall roughness	Surface height variation of via walls	AFM, SEM, white-light interferometry	Affects seed-layer coverage and electrical reliability
Sidewall verticality	Deviation of sidewall profile from ideal vertical geometry	Cross-sectional SEM, taper-angle measurement	Influences filling uniformity and current distribution
Diameter uniformity	Variation in via diameter among arrays	SEM image analysis	Reflects process consistency and fabrication yield
Etching defects	Cracks, residues, bubbles, and profile abnormalities	SEM, optical microscopy	Affects structural integrity and reliability

**Table 2:** Comparison of representative glass systems used in TGV wet etching and related glass micromachining.

Glass System	Representative Characteristics	Etching/Morphology Behavior	Main Limitation
Soda-lime glass [37]	High content of network modifiers	Near-isotropic dissolution in HF-based systems	Difficult lateral-etch control
Borosilicate glass [37]	Multicomponent silicate network	Composition-dependent dissolution and local passivation	Strong dependence on glass formulation
Fused silica [17,20]	SiO_2_-dominated network	Suitable for laser-induced selective etching with high modified/unmodified selectivity	Requires laser modification for efficient directional processing
Photosensitive glass [38]	Photosensitive multicomponent glass	Selective etching after exposure and thermal treatment	Restricted to specific material systems

**Table 3:** Comparison of representative TGV wet-etching studies and related glass micromachining processes.

Study/Route	Glass/Structure	Etchant/Assistance	Key Metric (s)	Main Morphology Outcome	Evidence Type
Ding et al. [14]	Borofloat 33/TGV	49% HF; reinforced Cr/Au mask	Taper angle ≈ 45°–50°	Pronounced undercutting and tapered sidewalls	TGV-specific
Konstantinova et al. [17]	Fused silica/test structure	BOE; varied NH_4_F/HF ratio	Etch rate and isotropy varied with composition	Transition among non-isotropic, isotropic, and undercutting regimes	Related glass microstructure
Kawaguchi et al. [18]	Silica glass/microtrench	LIBWE	AR ≈ 60	High-AR deep microstructure formation	Related glass microstructure
Fang et al. [19]	Glass/TGV	Optimized LIWE	Roughness reduced from 1.257 μm to 25 nm	Significant improvement in sidewall quality	TGV-specific
Liu et al. [20]	Fused silica/TGV	Femtosecond laser + selective wet etching	AR 11.7–61.3; ≈10,000 TGVs/min	High AR combined with high throughput	TGV-specific
Hou et al. [36]	Corning Eagle XG/TGV array	Ultrasound + temperature control + substrate motion	>26,000 TGVs; Diameter RSD < 1%	Improved large-array consistency	TGV-specific
Mu et al. [39]	Glass/microchannel	HF + laminar-flow confinement	AR ≈ 1	Suppressed lateral etching	Related glass microstructure
Zhang et al. [45]	Glass/TGV	PDDA-assisted LIWE	AR 23:1; verticality ≈ 0.77	Improved directionality and sidewall profile	TGV-specific
Yook et al. [38]	Photosensitive glass/TGV	Exposure + thermal treatment + selective etching	AR ≈ 4:1	Selective via formation with good sidewall quality	TGV-specific
Lee et al. [47]	Glass/TGV	Laser modification + wet chemical etching	AR ≈ 10:1; taper angle ≈ 2°	Straight, hourglass, and tapered profiles	TGV-specific

Related glass microstructures are included as mechanistic or process-related evidence rather than as direct TGV performance benchmarks.

## Data Availability

No new data were created or analyzed in this study. Data sharing is not applicable to this article.

## Abbreviations

The following abbreviations are used in this manuscript:

AR	Aspect ratio
CTE	Coefficient of thermal expansion
HTHH	High-temperature high-humidity
LAWE	Laminar flow-assisted wet etching
LIBWE	Laser-induced backside wet etching
LIDE	Laser-induced deep etching
LIWE	Laser-induced wet etching
MMIC	Monolithic microwave integrated circuit
RLGC	Resistance-inductance-conductance-capacitance
RSD	Relative standard deviation
SEM	Scanning electron microscopy
SLE	Selective laser-induced etching
TGV	Through-glass via
TSV	Through-silicon via
ULE	Ultra-low expansion
BHF	Buffered hydrofluoric acid
BOE	Buffered oxide etchant
TMAH	Tetramethylammonium hydroxide
PDDA	Poly(diallyldimethylammonium chloride)
IPA	Isopropyl alcohol
SDS	Sodium dodecyl sulfate
